# Morphological, Physiological, and Photosynthetic Differences of Tartary Buckwheat Induced by Post-Anthesis Drought

**DOI:** 10.3390/plants13152161

**Published:** 2024-08-05

**Authors:** Hang Yuan, Qiang Wang, Anyin Qi, Shuang Li, Yan Hu, Zhiming Hu, Laichun Guo, Chenggang Liang, Wurijimusi Li, Changying Liu, Yanxia Sun, Liang Zou, Lianxin Peng, Dabing Xiang, Cheng Liu, Jingwei Huang, Yan Wan

**Affiliations:** 1 Key Laboratory of Coarse Cereal Processing, Ministry of Agriculture and Rural Affairs, Sichuan Engineering & Technology Research Center of Coarse Cereal Industrialization, College of Food and Biological Engineering, Chengdu University, Chengdu 610106, China; yuanhang@stu.cdu.edu.cn (H.Y.); wangqiangeternity@icloud.com (Q.W.); qianyin@stu.cdu.edu.cn (A.Q.); lishuang27@stu.cdu.edu.cn (S.L.); huyan@stu.cdu.edu.cn (Y.H.); 18482188126@163.com (Z.H.); liuchangying@cdu.edu.cn (C.L.); sunyanxia1976@cdu.edu.cn (Y.S.); zouliangcdu@126.com (L.Z.); penglianxin@cdu.edu.cn (L.P.); dabing.xiang@163.com (D.X.); 2 Agronomy College, Jilin Agricultural University, Changchun 130118, China; 3 Baicheng Academy of Agricultural Sciences, No. 17, Sanhe Road, Taobei District, Baicheng 137000, China; guolaichun@126.com; 4 Sichuan Institute of Food Inspection, Chengdu 610097, China; 5 Research Center of Buckwheat Industry Technology, School of Life Sciences, Guizhou Normal University, Guiyang 550001, China; jesselcg@163.com; 6 Hinggan League Institute of Agricultural and Animal Husbandry Sciences, Hinggan League 137400, China; jimusi1117@163.com; 7 College of Agronomy and Horticulture, Chengdu Agricultural College, Chengdu 611130, China; 8 Chongqing Field Scientific Observation and Research Station for Authentic Traditional Chinese Medicine in the Tree Gorges Reservoir Area, College of Biological and Chemical Engineering, Chongqing University of Education, Chongqing 400067, China; 9 School of Preclinical Medicine, Chengdu University, Chengdu 610106, China

**Keywords:** Tartary buckwheat, drought stress, post-anthesis drought, morphological trait, physiology and biochemistry

## Abstract

Tartary buckwheat (*Fagopyrum tataricum* (L.) Gaertn) is a crop of significant interest due to its nutritional value and resilience to drought conditions. However, drought, particularly following flowering, is a major factor contributing to yield reduction. This research employed two distinct Tartary buckwheat genotypes to investigate the effects of post-anthesis drought on growth and physicochemical characteristics. The study aimed to elucidate the response of Tartary buckwheat to drought stress. The findings indicated that post-anthesis drought adversely impacted the growth, morphology, and biomass accumulation of Tartary buckwheat. Drought stress enhanced the maximum photosynthetic capacity (Fv/Fm) and light protection ability (NPQ) of the ‘Xiqiao-2’ genotype. In response to drought stress, ‘Dingku-1’ and ‘Xiqiao-2’ maintained osmotic balance by accumulating soluble sugars and proline, respectively. Notably, ‘Xiqiao-2’ exhibited elevated levels of flavonoids and polyphenols in its leaves, which helped mitigate oxidative damage caused by drought. Furthermore, rewatering after a brief drought period significantly improved plant height, stem diameter, and biomass accumulation in ‘Dingku-1’. Overall, ‘Xiqiao-2’ demonstrated greater long-term tolerance to post-anthesis drought, while ‘Dingku-1’ was less adversely affected by short-term post-anthesis drought.

## 1. Introduction

Tartary buckwheat (*Fagopyrum tataricum* (L.) Gaertn) is an annual herb known for its richness in mineral elements, protein, resistant starch, and particularly phenols. This plant exhibits significant biological activity and has been linked to the prevention of various chronic human diseases, including obesity, hypertension, and cardiovascular diseases [1]. Originally hailing from the mountains near the Himalayas in southwestern China, Tartary buckwheat has demonstrated remarkable adaptability to harsh environmental conditions such as high temperatures and drought [2]. Despite its tolerance, drought remains a key limiting factor for the development of the Tartary buckwheat industry in arid and semi-arid regions.

Drought is the most important environmental constraint on global crop productivity and food security [3]. Lack of water has a great impact on plant growth, survival, physiology, and yield [4]. About 80–95% of the fresh biomass of plants is composed of water, which is essential for various physiological processes such as plant growth, development, and metabolism [5]. Drought alters plant water usage, hinders stomatal movement, affects respiration and photosynthesis, disrupts cellular redox reactions, and causes peroxidation damage [6]. Photosynthesis is crucial for plant growth and metabolism, and water deficiency can reduce electron transport efficiency and H^+^ supply and lead to photosynthetic organ degradation [7]. Plants employ strategies like drought escape, drought avoidance, and drought tolerance [8]. Drought-tolerant plants respond to drought by enhancing root growth, regulating stomata, synthesizing osmotic substances, and activating cellular antioxidant systems [9].

The flowering stage, recognized as a water-sensitive period, significantly affects biomass distribution between the vegetative and filling stages. Drought stress during this critical period can profoundly influence plant growth and grain-filling processes [10]. This stage marks an essential transition for plants from vegetative to reproductive growth. The reduction in physiological grain yield under post-anthesis drought stress primarily results from the interruption of photosynthesis at this stage [11]. This phenomenon is crucial for supplying the assimilates necessary for grain filling [12]. A study by Verbeke et al. on wheat demonstrated that the osmotic pressure in stems and flag leaves remained low for an extended period following post-anthesis drought. Recovery after rewatering was more challenging compared to pre-anthesis drought, indicating that more energy was devoted to osmotic regulation during the post-anthesis phase than during the pre-anthesis phase [13].

Tartary buckwheat is a subject of significant research interest due to its high nutritional content and ability to withstand stress. However, existing studies have primarily focused on the impact of PEG-simulated osmotic stress on gene expression in Tartary buckwheat seedlings [14,15,16], neglecting the effects of drought stress during the flowering and filling stages. This study selected two Tartary buckwheat cultivars with distinct genotypes for investigation, conducting a pot water control experiment to assess the effects of post-anthesis drought and subsequent rewatering on growth, physiology, and yield. The aim was to compare the responses of different cultivars to varying stages of drought stress and rewatering, as well as to understand the recovery mechanism of drought tolerance of Tartary buckwheat. This research sets the groundwork for future investigations into the differences in drought tolerance among Tartary buckwheat varieties.

## 2. Results

### 2.1. Plant Morphological Traits

**Figure 1:**
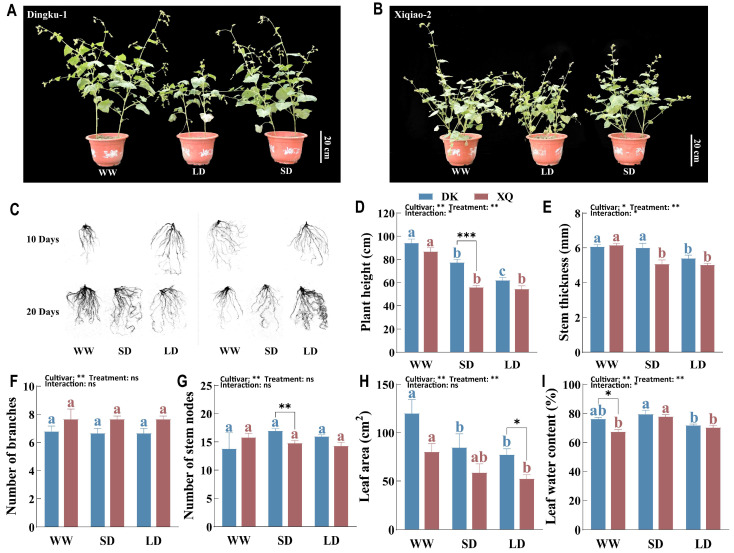
Analysis of growth morphology of Tartary buckwheat under drought stress. (**A**,**B**) Plant growth phenotype of DK and XQ. (**C**) Root morphology after 10 and 20 days of drought treatment. (**D**) Plant height. (**E**) Stem thickness. (**F**) Number of branches. (**G**) Number of stem nodes. (**H**) Leaf area. (**I**) Leaf water content. All data, except for root morphology, were collected over a 20-day period of drought. DK and XQ refer to the two Tartary buckwheat cultivars, “Dingku-1” and “Xiqiao-2”, respectively. WW, SD, and LD denote the three treatments: normal watering, short-term drought stress, and long-term drought stress, respectively. Duncan’s multiple-range test compared the means. Significant differences (*p* < 0.05) are marked with different letters (different treatments of the same cultivar are represented by the same color, blue or red). *, **, and *** represent significant differences at *p* < 0.05, *p* < 0.01, and *p* < 0.001 under two-way ANOVA and *t*-tests of different cultivars in the same treatment, respectively. ns, not significant. The results were presented as mean ± SEM (*n* = 5).

The study documented plant morphology (Figure 1A,B) and measured the total root length (TRL), total root surface area (RSA), root volume (RV), and average root diameter (RD) of two Tartary buckwheat cultivars on the 10th and 20th day of drought treatment (Table 1 and Figure 1C). Results showed that drought had varying effects on the two genotypes. After 10 days of drought, TRL, RSA, and RD increased in one cultivar (DK) and decreased in the other (XQ) under drought stress, although the changes were not statistically significant. And DK had significantly developed roots (TRL, RSA, and RD) compared to XQ. Interestingly, by the 20th day, TRL, RSA, and RD decreased in DK and increased in XQ. DK and XQ had longer roots in short-term drought (rewatering after drought) and long-term drought, respectively. There was a significant interaction between cultivars and treatments, with notable differences between the two cultivars.

The aboveground morphology of both cultivars exhibited similar responses to drought (Figure 1D–G), characterized by a significant reduction in plant height and stem diameter after 20 days of drought exposure. Upon restoration of the water supply following a short-term drought, notable differences in plant height emerged between the two cultivated species, with cultivar DK demonstrating less susceptibility to drought effects. Furthermore, drought conditions resulted in a decrease in the number of primary stem nodes in cultivar XQ and inhibited stem elongation. Overall, the differences in aboveground morphology between the two cultivars were minimal; however, a significant interaction between cultivar and drought treatment was observed concerning plant height and stem diameter. Drought conditions markedly decreased leaf area in both cultivars, particularly in XQ, while rewatering tended to enhance leaf yield following the drought period. Furthermore, long-term drought resulted in decreased leaf water content (LWC) in DK; however, LWC increased significantly in both cultivars under short-term drought stress (Figure 1H,I).

### 2.2. Chlorophyll Content and Chlorophyll Fluorescence Parameters

**Figure 2:**
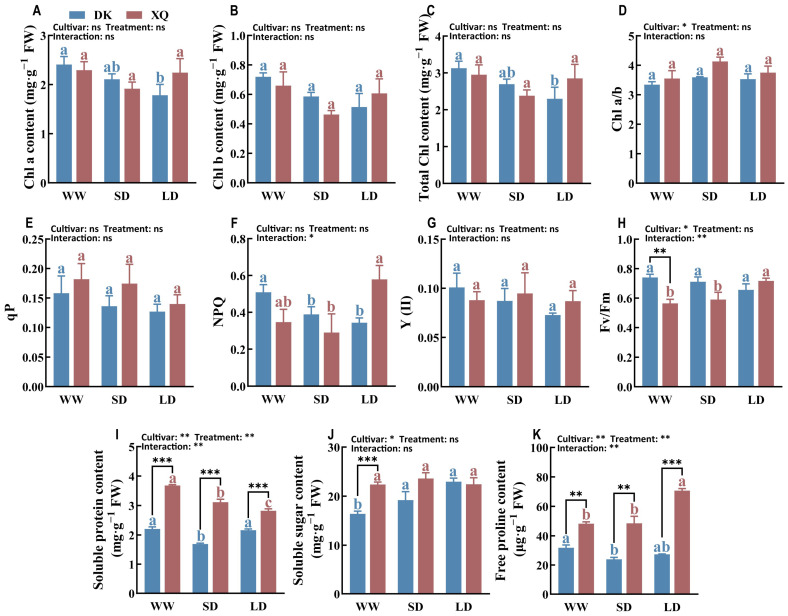
Analysis of photosynthetic characteristics and osmotic regulation of Tartary buckwheat under drought stress. (**A**) Chlorophyll a content. (**B**) Chlorophyll b content. (**C**) Total chlorophyll content. (**D**) Chlorophyll a/b. (**E**) Photochemical quenching coefficient. (**F**) Non-photochemical quenching coefficient. (**G**) The photochemical quantum yield of photosystem II. (**H**) The maximum photochemical quantum yield of PS II. (**I**) Soluble protein content. (**J**) Soluble sugar content. (**K**) Free proline content. DK and XQ refer to the two Tartary buckwheat cultivars, “Dingku-1” and “Xiqiao-2”, respectively. WW, SD, and LD denote the three treatments: normal watering, short-term drought stress, and long-term drought stress, respectively. Duncan’s multiple-range test compared the means. Significant differences (*p* < 0.05) are marked with different letters (different treatments of the same cultivar are represented by the same color, blue or red). *, **, and *** represent significant differences at *p* < 0.05, *p* < 0.01, and *p* < 0.001 under two-way ANOVA and *t*-tests of different cultivars in the same treatment, respectively. ns, not significant. The results were presented as mean ± SE (*n* = 5).

The chlorophyll content and chlorophyll fluorescence of two cultivars of Tartary buckwheat were analyzed (Figure 2A–H). Drought conditions resulted in a significant increase in NPQ and Fv/Fm values for the XQ cultivar, thereby enhancing the light protection and energy conversion efficiency of its leaves. In contrast, drought negatively affected the Chl a content, Chl b content, total Chl content, Y (II), qP, NPQ, and Fv/Fm parameters of the DK cultivar, with these effects becoming more pronounced as the duration of drought increased. Additionally, the chlorophyll a/b ratio for both DK and XQ cultivars increased following drought stress, with a notable rise observed under short-term drought conditions.

### 2.3. Osmotic Regulation Response

Osmotic regulation in plants involves responses to environmental factors such as drought. The levels of soluble protein, soluble sugar, and free proline were measured in two cultivars of Tartary buckwheat (Figure 2I,J). The two cultivars exhibited distinct patterns of osmotic accumulation under arid conditions, with cultivar XQ showing higher levels of soluble protein, soluble sugar, and free proline. Drought stress during the flowering stage resulted in a significant increase in soluble sugar accumulation in cultivar DK and free proline accumulation in cultivar XQ leaves, while soluble protein levels decreased significantly.

### 2.4. Antioxidant Activity

**Figure 3:**
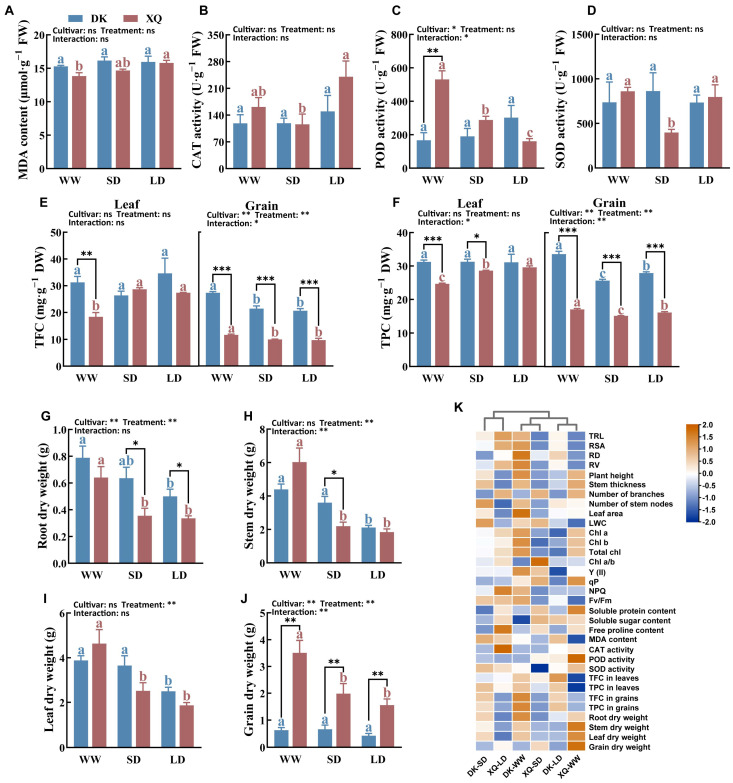
Analysis of antioxidant activities and biomass of Tartary buckwheat leaves under drought stress. (**A**) Malondialdehyde (MDA) content. (**B**) Catalase (CAT) activity. (**C**) Peroxidase (POD) activity. (**D**) Superoxide dismutase (SOD) activity. (**E**) Content of total flavonoids in leaf and grain. (**F**) Total polyphenols content in leaf and grain. (**G**–**J**) Represent the dry weight of the root, stem, leaf, and grain with different durations of drought stress, respectively, which are the average values of individual plant data. (**K**) Heat-map analysis. DK and XQ refer to the two Tartary buckwheat cultivars, “Dingku-1” and “Xiqiao-2”, respectively. WW, SD, and LD denote the three treatments: normal watering, short-term drought stress, and long-term drought stress, respectively. Duncan’s multiple-range test compared the means. Significant differences (*p* < 0.05) are marked with different letters. Treatments of the same cultivar are represented by the same color (blue or red). The lowercase letters in TFC and TPC denote significant differences only between different cultivars and treatments within the same vegetative organ. *, **, and *** represent significant differences at *p* < 0.05, *p* < 0.01, and *p* < 0.001 under two-way ANOVA and *t*-tests of different cultivars in the same treatment, respectively. ns, not significant. The results were presented as mean ± SE (*n* ≥ 4).

Under drought stress, the levels of malondialdehyde (MDA) in XQ leaves increased significantly and were positively correlated with the duration of drought. While drought stress slightly elevated the activities of catalase (CAT) and peroxidase (POD) in DK, it significantly decreased the POD activity in XQ. Notably, CAT activity exhibited a more pronounced change following long-term drought compared to short-term drought. Under normal watering conditions, XQ demonstrated higher POD activity than DK (Figure 3A–D).

Flavonoids and polyphenols exhibit potent antioxidant properties. Following drought treatment, total flavonoid content (TFC) and total polyphenol content (TPC) levels in XQ leaves showed a significant increase, while no notable differences were observed in the grains. In contrast, drought did not induce significant changes in TFC and TPC levels in DK leaves; however, it did lead to a significant reduction in the contents of these compounds in their grains. The accumulation of flavonoids and polyphenols varies between the two Tartary buckwheat cultivars, with DK exhibiting significantly higher levels in both leaves and seeds compared to XQ, particularly in seeds where the difference between cultivars is nearly double (Figure 3E,F).

### 2.5. Biomass Accumulation

Water is an essential raw material for plant growth and organic synthesis. In this study, we measured the biomass of roots, stems, leaves, and grains of Tartary buckwheat after 20 days of drought (Figure 3G–J). We found that the impact of drought on biomass accumulation was similar across two different genotypes of Tartary buckwheat. Drought significantly inhibited dry matter accumulation in roots, stems, leaves, and grains. Although XQ exhibited faster grain filling, it was considerably affected by drought stress during the flowering stage. Despite being slower to respond to rewatering compared to DK, XQ was unable to fully recover from the effects of drought during the flowering stage, even with irrigation.

## 3. Discussion

The response of plants to drought is influenced by the duration and severity of stress factors, along with the genetic characteristics related to stress response capabilities [17]. Research on drought stress has consistently been a central focus in agricultural studies. This study aims to investigate the variations in growth morphology, reactive oxygen species (ROS) detoxification, and photosynthetic performance among different genotypes of Tartary buckwheat during the flowering stage under drought stress.

### 3.1. Effect of Post-Anthesis Drought on Growth Morphology of Tartary Buckwheat

The impact of water deficit on plants is evident through various observable changes, including decreased plant height, leaf wilting, and alterations in leaf number and area. Root morphology and physiology play a crucial role in determining aboveground growth and overall plant yield, as roots are responsible for the absorption of water and nutrients from the soil [18]. Previous research has indicated that drought stress can lead to increased root density, reduced root diameter, accelerated death of fine roots, and a negative correlation between root hair lifespan and drought stress [19]. Linear increases in root length, surface area, and volume have been observed in wheat [20] and soybean [21] under drought conditions. This study analyzed the root morphology of two different genotypes of Tartary buckwheat, revealing contrasting responses to drought. Early drought stress during the post-anthesis stage stimulated root growth in DK while inhibiting it in XQ. Conversely, long-term drought inhibited root growth in DK, potentially due to the death of slender roots resulting from prolonged water scarcity, while XQ exhibited a delayed root-system response to drought. Previous research has demonstrated significant reductions in plant height under drought stress across various crops, including lily [22], sugarcane [23], corn [24], and rice [25]. This phenomenon is also observed in the tropical crop banana [26]. Similarly, both genotypes of Tartary buckwheat experienced a notable decrease in plant height under drought stress in this study, with only DK exhibiting aboveground growth restoration following short-term drought and subsequent re-watering. These findings indicate that post-anthesis drought adversely affects root and aboveground growth in Tartary buckwheat, with DK displaying a more rapid response to drought compared to XQ. Leaves, as the primary organs for assimilation and transpiration, play a crucial role in plant photosynthesis and yield. Alterations in leaf area directly impact these processes, rendering it a key observable characteristic of plant leaves under drought stress [27]. Research has shown that the reduction in leaf area during drought stress is primarily linked to decreased leaf swelling and photosynthetic rate, with variations depending on crop variety [28]. Both genotypes of Tartary buckwheat experienced significant decreases in leaf area under drought stress; however, DK demonstrated greater sensitivity to short-term drought conditions. Furthermore, DK’s leaf water content (LWC) varied in response to changes in soil moisture, indicating its capacity to detect fluctuations in soil moisture levels and promptly adjust its water conservation strategies.

### 3.2. Effects of Post-Anthesis Drought on Photosynthesis of Tartary Buckwheat

Photosynthesis is a crucial physiological process in plants and serves as a key indicator of drought stress due to its high sensitivity to environmental changes [29]. Drought stress is also a significant indicator of damage to photosynthetic pigments, leading to a reduction in chlorophyll content [30,31]. The decrease in chlorophyll content observed in both Tartary buckwheat cultivars under drought stress, as illustrated in Figure 1, corroborates previous research findings. Notably, the ratio of chlorophyll a/b increased during this period, particularly in the XQ cultivar following rehydration post-drought. This observation suggests that the effects of drought and subsequent rehydration on the photosynthetic reaction centers (RCs) are less pronounced than on the light-harvesting complex II (LHCII), which contains chlorophyll b. Chlorophyll fluorescence has been established as a valuable non-invasive method for assessing the inhibition of PSII electron transport resulting from damage in drought stress studies [32]. Some studies indicate that severe drought stress can lead to photoinhibition quenching of PSII RC, typically characterized by a reduction in Fv/Fm [33]. This study found that DK exhibited a decrease in both photosynthetic and photoprotection capacities under drought stress, whereas XQ demonstrated higher Fv/Fm and NPQ levels. Previous research indicated that Fv/Fm significantly decreases only when plant water storage falls below a critical level (<20% RWC) [29]. In certain plant species, such as peas [34] and triticale [35], severe drought stress did not result in a notable change in Fv/Fm. Arabidopsis thaliana responds to short-term stress by reducing photochemical efficiency through increased NPQ levels [36]. Prolonged drought stress leads to a significant increase in NPQ levels [37]. The study indicates that post-anthesis drought negatively impacts the photosynthesis of Tartary buckwheat, resulting in decreased biomass and impairing the filling process. Notably, XQ exhibited greater drought tolerance than DK, as the current level of drought stress did not exceed XQ’s critical tolerance threshold. Furthermore, a moderate water deficit environment was found to enhance the leaf photosynthetic capacity of XQ.

### 3.3. Physiological Response of Tartary Buckwheat to Post-Anthesis Drought

Drought stress results in a reduction in available water for plants, leading to a loss of water from plant cells. To combat the osmotic pressure induced by water scarcity, plants typically reduce intracellular water, decrease cell volume, and increase cell contents [38]. The osmotic substances found within plants primarily consist of organic osmotic substances and inorganic ions, characterized by low molecular weight, high solubility, and low cytotoxicity [27]. In this study, it was observed that DK plants accumulated soluble sugar when subjected to drought stress, whereas XQ plants tended to accumulate free proline. Previous research has indicated that the Lanzhou lily can withstand drought conditions by decreasing the levels of soluble sugars, polysaccharides, and fructose while increasing proline and glucose levels to regulate osmotic balance and metabolism [22]. Furthermore, additional studies have shown that soluble carbohydrates, such as sucrose and glucose, increase during short-term drought conditions but significantly decrease after prolonged severe drought, accompanied by a notable rise in proline and mannitol levels [39,40,41].

Our research revealed that the MDA content of Tartary buckwheat increased in both cultivars following exposure to drought stress. Drought stress is known to trigger the excessive accumulation of reactive oxygen species (ROS), which leads to peroxidation damage in plant cells [42]. To mitigate the harmful effects of ROS accumulation, plants have evolved various enzymatic and non-enzymatic antioxidant systems. Antioxidant enzymes such as CAT, POD, and SOD play a crucial role in ROS scavenging; thus, enhancing the activity of these enzymes can alleviate oxidative stress induced by environmental factors [43]. Our study observed a significant increase in CAT enzyme activity in the two Tartary buckwheat genotypes, which primarily contributed to ROS scavenging. Rehydration following drought stress was found to restore the MDA content and antioxidant enzyme activities in both Tartary buckwheat cultivars to normal levels, underscoring the importance of water in facilitating ROS scavenging and supporting antioxidant enzyme function within plant cells. Interestingly, the activity of POD was slightly increased in DK but significantly decreased in XQ under drought stress. A previous study indicated that short-term drought did not lead to significant changes in POD activity, whereas long-term drought lasting more than 20 days significantly reduced its activity [44]. This decrease in POD activity may result from severe oxidative damage caused by prolonged drought, rendering POD less effective in responding to drought stress. Furthermore, non-enzymatic antioxidants play a crucial role in alleviating oxidative stress in plants. Phenolic compounds are known for their potent antioxidant properties due to their unique molecular structure, and prior research has documented an increase in phenolic accumulation in plants subjected to drought conditions [21,45,46]. Our findings revealed a significant increase in flavonoids and polyphenols in the leaves of XQ, while a notable decrease was observed in the grains of DK during drought stress. The accumulation patterns of flavonoids and polyphenols varied across different organs of the two cultivars, with XQ preferentially accumulating these compounds in its leaves to combat drought stress and delay leaf senescence. In contrast, DK grains exhibited higher levels of flavonoids and polyphenols that decreased under drought stress, suggesting a disruption in the transport of these compounds from leaves to grains in DK during drought conditions.

### 3.4. Effects of Drought Post-Anthesis on Biomass Accumulation of Tartary Buckwheat and Membership Function Analysis

Crops frequently experience drought conditions, leading to significant reductions in overall yield. In agricultural terms, a drought-tolerant plant is defined as one that can sustain crop production during periods of gradual and moderate soil moisture deficits, often without exhibiting protective mechanisms [47]. Most crop varieties demonstrate heightened sensitivity to water deficits, particularly during the flowering stage; in the event of water shortages, grain abortion may occur, resulting in considerable yield losses. This study reveals that under drought stress, the root, stem, leaf, and grain biomass of the drought-tolerant plant Tartary buckwheat were adversely affected, with drought significantly influencing biomass and yield formation. Yield formation is primarily dependent on the accumulation, distribution, and transport of dry matter, which is largely derived from photosynthesis—the ultimate product of photosynthetic processes [48]. Drought stress diminishes the photosynthetic rate during the grain-filling period, impedes the synthesis of photosynthetic products post-flowering, and restricts the transport of these products to seeds prior to flowering, ultimately leading to reduced yield [49]. Furthermore, this study indicates that the decline in photosynthetic pigment content, along with differential changes in fluorescence parameters, significantly impacts the biomass accumulation of various plant organs. Additionally, water deficiency obstructs material transport, alters osmotic pressure, and causes the decomposition of most organic matter into smaller molecular substances that are utilized to equilibrate intracellular and extracellular osmotic pressure, thereby further constraining biomass accumulation.

Heat-map analyses were conducted on Tartary buckwheat following 20 days of post-anthesis drought (Figure 3K). The XQ cultivar demonstrated tolerance to prolonged drought conditions through enhanced root growth, increased photosynthetic capacity, and increased antioxidant enzyme activity. In contrast, the DK variety exhibited greater susceptibility to drought stress, characterized by a significant accumulation of flavonoids under stress conditions. Weighted membership function analysis (Table 2 and Table 3) revealed that DK displayed improved morphological and physiological characteristics upon rehydration after drought, surpassing those observed under normal irrigation. The duration of drought had a more pronounced impact on DK, whereas XQ showed greater adaptability to long-term drought stress, albeit with lower plasticity.

## 4. Materials and Methods

### 4.1. Plant Material and Growing Conditions

This study focused on two commonly used cultivars in the field, ‘Dingku-1’ and ‘Xiqiao-2’, referred to hereafter as ‘DK’ and ‘XQ’, respectively. Prior to sowing, uniform and plump seeds were sterilized with a 1% sodium hypochlorite solution for 15 min, followed by three washes with distilled water. The sterilized seeds were then evenly sown in plastic pots filled with nutrient-rich soil that had been pre-soaked with water. The dimensions of the plastic pots are as follows: upper outer diameter of 26.5 cm, upper inner diameter of 23 cm, bottom diameter of 15.7 cm, and height of 17.6 cm. Approximately 15 seeds were sown in each pot, and the pots were watered daily. Once the Tartary buckwheat seedlings reached the three-leaf and one-core stage, there were 2 to 3 seedlings per pot. The soil used in the experiment contained 17.3 g·kg^−1^ of organic matter, 1.05 g·kg^−1^ of total nitrogen, 0.813 g·kg^−1^ of total phosphorus, 1.96 g·kg^−1^ of total potassium, 63 mg·kg^−1^ of alkaline-hydrolyzed nitrogen, 31.4 mg·kg^−1^ of available phosphorus, and 87 mg·kg^−1^ of fast-acting potassium. Basal fertilizer was applied at the time of sowing (300 kg·ha^−1^; N 15%, P_2_O_5_ 15%, K_2_O 15%), and urea (100 kg·ha^−1^; 46% N) was applied when the Tartary buckwheat began to bloom.

### 4.2. Control of Drought Conditions

**Figure 4:**
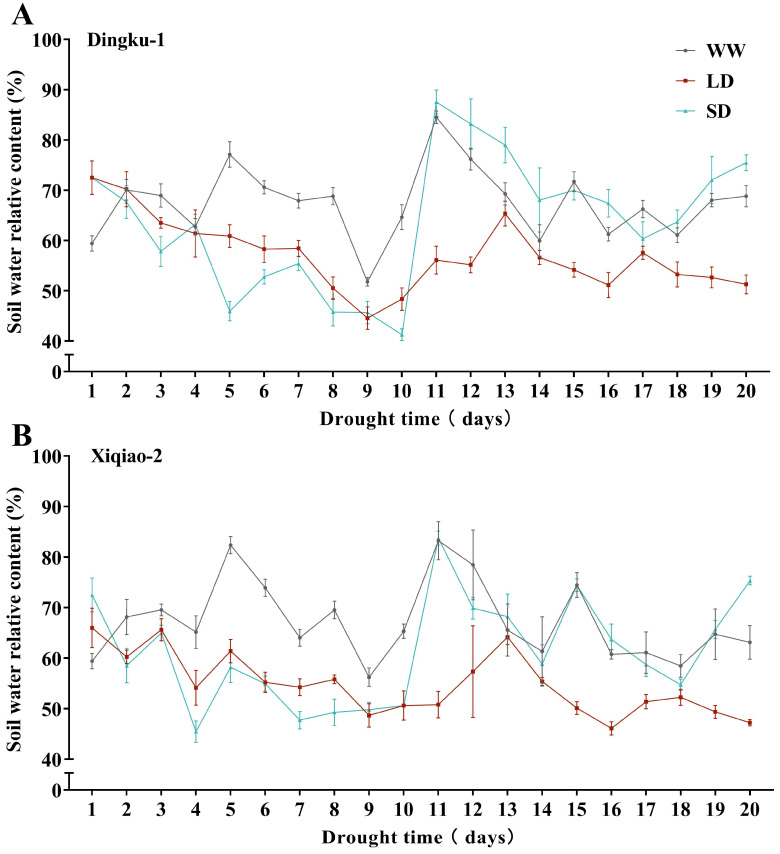
Dynamics of soil relative water content during normal watering, drought treatment, and rewatering. (**A**) Soil relative water content dynamics of “Dingku-1” cultivated species. (**B**) Soil relative water content dynamics of “Xiqiao-2” cultivation. WW, SD, and LD denote the three treatments: normal watering, short-term drought stress, and long-term drought stress, respectively.

To simulate post-anthesis drought stress, we conducted a 20-day drought treatment during the bloom period through artificial irrigation. The experiment included three treatments: normal watering (WW), which maintained soil relative water content at 70–80% for 20 days; short-term drought stress (SD), which involved drought stress for the first 10 days followed by normal watering for the subsequent 10 days; and long-term drought stress (LD), where soil relative water content was maintained at 40–50% for 20 days. Soil volumetric water content was measured daily after sunset (approximately 17:00) using a soil moisture meter (WET-2, Delta-T Devices Ltd., Cambridge, UK). Soil relative water content was calculated, and treatments that fell below the moisture control threshold were irrigated (measured volumetric water content divided by field water holding capacity, with the average field water holding capacity determined in this study being 49.38%). Soil water content dynamics were recorded, as shown in Figure 4, and samples of Tartary buckwheat were collected on the 10th and 20th days of treatment, respectively.

### 4.3. Plant Growth, Morphological Parameters, and Biomass

Tartary buckwheat samples were collected following a 20-day drought treatment, during which the plant height and stem diameter of various cultivars were measured using a ruler and a vernier caliper. The dry matter mass of the roots, stems, leaves, and effectively filled seeds (including full grains and discolored seed coats from the main stem and branches after winnowing) of each Tartary buckwheat plant was determined using an analytical balance. The root morphology of Tartary buckwheat was scanned and analyzed using WinRHIZO Pro 32-bit 2017a software (Version 2007d, Regent Instrument Inc., Quebec, QC, Canada).

The leaf area of Tartary buckwheat was measured and calculated using a 1 cm^2^ leaf area punch. The relative water content of the leaves was adjusted according to the methods described by Barrs and Weatherly [50]. The leaves from the 7th, 8th, and 9th nodes of the main stem of Tartary buckwheat (functional leaves [51]) from bottom to top were cut into pieces and immediately weighed to determine the fresh weight (FW). Subsequently, the samples were immersed in a petri dish filled with water and refrigerated at +10 °C for 24 h. After excess water was removed and the expansion mass (TW) was measured, the samples were dried in an oven at 80 °C for 24 h to obtain the dry weight (DW).

The relative water content of the leaf was calculated using the formula:

LWC (%) = [(FW − DW)/(TW − DW)] × 100%

### 4.4. Chlorophyll Content and Chlorophyll Fluorescence

Leaves from nodes 7, 8, and 9 were sampled, chopped, and mixed. A 0.1 g sample was soaked in 5 mL of 95% ethanol for 24 h in darkness to ensure complete decolorization. Following the method outlined by Arnon [52], the levels of chlorophyll a (Chl a) and chlorophyll b (Chl b) in the supernatant were determined using a spectrophotometer (UV2600A, UNICO Shanghai China) at wavelengths of 665 nm and 649 nm.

Chlorophyll fluorescence parameters were measured using a portable JUNIOR PAM device (WALZ, Effeltrich, Germany). Leaves from the 8th section of each plant were selected and subjected to a 0.5-h dark adaptation period prior to analysis. The maximum quantum yield of PSII (Fv/Fm), photochemical quantum yield of photosystem II [Y (II)], photochemical quenching (qP), non-photochemical quenching (NPQ), and the non-photochemical quenching coefficient (qN) were directly calculated using the WinControl-3 software.

### 4.5. Soluble Sugar, Soluble Protein, and Free Proline

The content of soluble sugars was quantified using the anthrone method [53], while the soluble protein content was determined through Coomassie Brilliant Blue G-250 staining [54]. The free proline content was assessed following the methodology described by Bates, Waldren, and Teare [55]. Specifically, 0.3 g of the chopped leaf sample was mixed with 3% sulfosalicylic acid and boiled in water for 10 min. Afterward, the supernatant was collected, and color development was achieved using ninhydrin. Following another round of boiling in a water bath, toluene extraction was performed, and the absorbance was measured at a wavelength of 520 nm.

### 4.6. MDA and Antioxidant Enzyme Activity

The antioxidant enzyme activities and malondialdehyde (MDA) contents of the collected Tartary buckwheat functional leaves were assessed. The activity of catalase (CAT) was evaluated using Aebi’s method [56], which measures the rate of hydrogen peroxide (H_2_O_2_) consumption per unit of enzyme extract per minute at a wavelength of 240 nm (U). The activity of superoxide dismutase (SOD) was determined at a wavelength of 560 nm, monitoring the photochemical reduction inhibition induced by nitro tetrazolium (NBT) [57]. One unit (U) of SOD activity is defined as the amount of enzyme required to achieve 50% inhibition of NBT reduction, as detected at 560 nm per hour. Peroxidase (POD) activity was measured using Velikova’s method at a wavelength of 470 nm [58]. One unit (U) of POD activity is defined as a change of 0.1 in absorbance at 470 nm per minute. The malondialdehyde (MDA) content in the leaves was quantified following Zhanassova’s method [59] and calculated using a specific formula:

MDA (μmol g^−1^FW) = 6.45(OD_532_ − OD_600_) − 0.56OD_450_.

### 4.7. Non-Enzymatic Antioxidants: Total Flavonoids, Total Polyphenols

The Tartary buckwheat leaves and grains were dried at 55 °C, ground into a fine powder, and sifted through a 60-mesh screen. A 0.1 g sample was then mixed with 5 mL of 80% methanol and subjected to ultrasonication at 50 °C for 30 min. The supernatant was collected via centrifugation, and the volume was adjusted to 5 mL with 30% methanol to prepare the extract. The total flavonoid content (TFC) was determined using aluminum chloride colorimetry, with rutin as the reference standard [60]. The total polyphenol content (TPC) was measured using the Folin–Ciocalteu reagent, incorporating slight modifications to the standard method [61]. Specifically, a 0.5 mL aliquot of the 10-fold diluted extract was mixed with 2.5 mL of 2M Folin phenol reagent, followed by the addition of 2 mL of 7.5% sodium carbonate after a 5 min interval. The reaction mixture was allowed to stand for 2 h in the dark, and the TPC was quantified at a wavelength of 760 nm, utilizing Gallic acid as the reference standard.

### 4.8. Membership Function Analysis

To investigate the response of DK and XQ to post-anthesis drought, the membership function value (MFV) was extensively assessed through principal component weight analysis [62].

$$Wi=PCi/\sum_{i=1}^{n}PCi\;\mu \left(Xj\right)=(Xj-Xmin)/(Xmax-Xmin)\;MFV=\sum_{j=1}^{n}\left[\mu \left(Xj\right)\times \left(Wi\right)\right]$$

In the formula, *Wi* and *PCi* are the weight and contribution rate of the *i*-th comprehensive index among all comprehensive indexes, respectively. And *n* is the number of extracted principal components. *μ*(*Xj*) is the membership function value of the *j* comprehensive index, *Xj* is the *j* comprehensive index value, and *Xmin* and *Xmax* are the minimum and maximum values of the *j* comprehensive index, respectively.

### 4.9. Statistical Analyses

All results were obtained from three or more replicated experiments. Statistical analyses were performed using SPSS 25 software (SPSS Inc., Chicago, IL, USA) for two-way ANOVA, one-way ANOVA, and Duncan’s multiple range test (*p* < 0.05). Graphical analysis and multiple *t*-test analyses were conducted using GraphPad Prism 5.0 (GraphPad Software, La Jolla, CA, USA).

## 5. Conclusions

This study found that post-anthesis drought significantly restricted the growth of Tartary buckwheat, inhibited its photosynthetic capacity, and reduced its biomass accumulation. XQ exhibited higher levels of malondialdehyde (MDA) under drought stress, alleviating osmotic stress and peroxidation damage to leaves by accumulating soluble proteins, free proline, and increasing catalase (CAT) activity. Concurrently, root growth was enhanced to adapt to prolonged drought conditions and to maintain a high photosynthetic capacity. Conversely, DK accumulated greater amounts of sugars, flavonoids, and polyphenols to cope with osmotic stress and oxidative damage resulting from long-term drought. In summary, post-anthesis drought can severely impact the growth, physiological processes, and grain-filling of Tartary buckwheat. XQ demonstrates greater long-term tolerance to post-anthesis drought, whereas DK is less affected by short-term drought events. This study broadens the understanding of drought tolerance in Tartary buckwheat; however, the underlying molecular mechanisms of drought tolerance and strategies for enhancing the adaptability and resilience of Tartary buckwheat to drought conditions remain key areas for further research.

## Figures and Tables

**Table 1:** Morphological analysis of Tartary buckwheat root under drought stress.

Time	Treatment	Variety	TRL (cm)	RSA (cm^2^)	RV (cm^3^)	RD (mm)
10 Days	WW	DK	1460.25 ± 172.78 ab	184.93 ± 26.66 ab	0.4 ± 0.02 a	1.88 ± 0.32 a
		XQ	1465.46 ± 156.11 ab	158.37 ± 9.21 bc	0.36 ± 0.02 a	1.4 ± 0.11 ab
	SD or LD	DK	1922.41 ± 141.74 a	214.72 ± 15.55 a	0.36 ± 0.01 a	1.91 ± 0.15 a
		XQ	1017.69 ± 142.2 b	108.93 ± 16.11 c	0.34 ± 0.01 a	0.93 ± 0.15 b
	F value	Cultivars	0.012 *	0.002 **	0.117	0.002 **
		Treatment	0.964	0.588	0.147	0.291
		C × T	0.011 *	0.041 *	0.476	0.227
20 Days	WW	DK	2861.91 ± 121.3 a	344.41 ± 34.3 a	0.38 ± 0.02 a	3.33 ± 0.53 a
		XQ	1325.36 ± 130.32 b	128.01 ± 11.68 d	0.31 ± 0 c	0.99 ± 0.09 d
	SD	DK	2315.46 ± 190.89 a	225.37 ± 21.47 c	0.31 ± 0.01 c	1.75 ± 0.2 cd
		XQ	1281.99 ± 171.02 b	132 ± 17.4 d	0.33 ± 0.01 bc	1.09 ± 0.16 d
	LD	DK	2264.59 ± 417.92 a	241.72 ± 37.77 bc	0.35 ± 0.02 ab	2.06 ± 0.26 bc
		XQ	3053.14 ± 338.35 a	324.8 ± 42.12 ab	0.34 ± 0.01 bc	2.76 ± 0.42 ab
	F value	Cultivar	0.008 **	0.004 **	0.044 *	0.004 **
		Treatment	0.007 **	0.005 **	0.095	0.007 **
		Interaction	0.001 **	0.001 **	0.003 **	0.001 **

Note: Total root length (TRL), root surface area (RSA), root volume (RV), and root diameter (RD). C and T indicate cultivar and treatment, respectively. DK and XQ refer to the two Tartary buckwheat cultivars, “Dingku-1” and “Xiqiao-2”, respectively. WW, SD, and LD denote the three treatments: normal watering, short-term drought stress, and long-term drought stress, respectively. Duncan’s multiple-range test compared the means. Significant differences (*p* < 0.05) are marked with different letters. * and ** represent significant differences at *p* < 0.05 and *p* < 0.01 under two-way ANOVA, respectively. The results were presented as mean ± SE (standard error, *n* = 5).

**Table 2:** Factor analysis of each treatment under drought stress.

Index	PC1	PC2	PC3	Index	PC1	PC2	PC3
TRL	0.054	−0.041	0.099	Soluble protein content	−0.06	0.045	0.053
RSA	0.056	−0.035	0.097	Soluble sugar content	−0.067	−0.034	−0.014
RD	0.045	−0.023	0.031	Free proline content	−0.045	−0.012	0.137
RV	0.058	−0.028	0.093	MDA content	0.043	−0.076	−0.004
Plant height	0.041	0.087	−0.009	CAT activity	−0.021	−0.027	0.144
Stem thickness	0.037	0.086	−0.035	POD activity	−0.045	0.066	−0.043
Number of branches	−0.062	0.016	0.084	SOD activity	0.031	0.038	0.054
Number of stem nodes	0.054	0.043	−0.091	TFC in leaves	0.036	−0.076	−0.04
Leaf area	0.061	0.052	−0.029	TPC in leaves	0.055	−0.068	−0.027
LWC	0.029	−0.029	−0.086	TFC in grains	0.07	0.01	−0.054
Chl a	0.025	0.065	0.116	TPC in grains	0.07	0.004	−0.045
Chl b	0.041	0.068	0.091	Root dry weight	0.052	0.072	−0.031
Total chl	0.03	0.067	0.111	Stem dry weight	0.009	0.103	−0.004
Chl a/b	−0.058	−0.043	−0.018	Leaf dry weight	0.016	0.098	−0.038
Y (II)	0.012	0.041	0.044	Grain dry weight	−0.057	0.064	0.038
qP	−0.038	0.072	0.005	Variance contribution rate (%)	40.325	29.141	17.29
NPQ	0.036	−0.016	0.152	Accumulated contribution rate (%)	40.325	69.465	86.755
Fv/Fm	0.063	−0.037	0.062	Weight factors (%)	46.4815	33.5900	19.9297

Note: TRL refers to total root length (cm), RSA refers to total root surface area (cm^2^), RD refers to mean root diameter (mm), RV refers to root volume (cm^3^), LWC refers to relative leaf water content (%), Chl refers to chlorophyll, Y (II) refers to the photochemical quantum yield of photosystem II, qP refers to the photochemical light quenching coefficient, NPQ refers to the non-photochemical light quenching coefficient, and Fv/Fm refers to the maximum photochemical light quantum yield of photosystem II. Additionally, MDA refers to malondialdehyde, CAT refers to catalase, POD refers to peroxidase, SOD refers to superoxide dismutase, TFC refers to total flavonoid content, and TPC refers to total polyphenol content.

**Table 3:** Analysis of membership function of each treatment under drought stress.

Sample	X1	X2	X3	μ1	μ2	μ3	MFV	Rank
DK-WW	1.484	0.568	0.375	1.000	0.564	0.469	0.748	1
XQ-WW	−0.880	1.694	0.153	0.115	1.000	0.384	0.466	2
DK-SD	0.685	−0.008	−0.743	0.701	0.341	0.039	0.448	3
XQ-LD	−0.317	−0.889	1.756	0.326	0.001	1.000	0.351	4
DK-LD	0.214	−0.891	−0.846	0.525	0.000	0.000	0.244	5
XQ-SD	−1.186	−0.474	−0.695	0.000	0.161	0.058	0.066	6

## Data Availability

All datasets supporting the conclusions of this article are included within the article. If not included in the manuscript, they are available from the corresponding authors upon reasonable request.
